# Prediction of End-Of-Season Tuber Yield and Tuber Set in Potatoes Using In-Season UAV-Based Hyperspectral Imagery and Machine Learning

**DOI:** 10.3390/s20185293

**Published:** 2020-09-16

**Authors:** Chen Sun, Luwei Feng, Zhou Zhang, Yuchi Ma, Trevor Crosby, Mack Naber, Yi Wang

**Affiliations:** 1 Biological Systems Engineering, University of Wisconsin–Madison, Madison, WI 53706, USA; csun223@wisc.edu (C.S.); lfeng52@wisc.edu (L.F.); ma286@wisc.edu (Y.M.); 2 Key Laboratory of Spectral Imaging Technology, Xi’an Institute of Optics and Precision Mechanics, CAS, Xi’an 710119, China; 3 Horticulture, University of Wisconsin-Madison, Madison, WI 53706, USA; twcrosby@wisc.edu (T.C.); mrnaber@wisc.edu (M.N.); wang52@wisc.edu (Y.W.)

**Keywords:** hyperspectral imaging, machine learning, tuber yield, tuber set, unmanned aerial vehicles

## Abstract

Potato is the largest non-cereal food crop in the world. Timely estimation of end-of-season tuber production using in-season information can inform sustainable agricultural management decisions that increase productivity while reducing impacts on the environment. Recently, unmanned aerial vehicles (UAVs) have become increasingly popular in precision agriculture due to their flexibility in data acquisition and improved spatial and spectral resolutions. In addition, compared with natural color and multispectral imagery, hyperspectral data can provide higher spectral fidelity which is important for modelling crop traits. In this study, we conducted end-of-season potato tuber yield and tuber set predictions using in-season UAV-based hyperspectral images and machine learning. Specifically, six mainstream machine learning models, i.e., ordinary least square (OLS), ridge regression, partial least square regression (PLSR), support vector regression (SVR), random forest (RF), and adaptive boosting (AdaBoost), were developed and compared across potato research plots with different irrigation rates at the University of Wisconsin Hancock Agricultural Research Station. Our results showed that the tuber set could be better predicted than the tuber yield, and using the multi-temporal hyperspectral data improved the model performance. Ridge achieved the best performance for predicting tuber yield (R^2^ = 0.63) while Ridge and PLSR had similar performance for predicting tuber set (R^2^ = 0.69). Our study demonstrated that hyperspectral imagery and machine learning have good potential to help potato growers efficiently manage their irrigation practices.

## 1. Introduction

Potato (*Solanum tuberosum* L.) is an important staple food crop, and improving potato production is of great importance for ensuring food security for the increasing world population [1]. As a shallow-rooted crop, potato is often grown in soils with low to medium water-holding capacities, and it is extremely sensitive to water stress [2,3]. Therefore, irrigation is required for profitable tuber yield and quality. So far, no tools have been available for the potato growers to predict their final production using in-season information that is easily collectable. Thus, timely estimation of tuber production during the growing season is necessary to improve irrigation efficiency, reduce the potential of yield loss caused by water stress, and minimize the impacts of irrigation on the groundwater table [4].

Unlike the traditional yield assessment method, the advanced remote sensing technique is based on a non-destructive and efficient approach which has great potential for estimating the crop production and monitoring the growth status over the growing season. In the past few decades, satellite data have been broadly used in crop yield prediction with various spatial and spectral resolution [5,6,7], but their adoption in precision farming is hampered by the nonnegligible cloud contamination and the poor spatial resolution [8]. Recently, the development of sensors promoted the application of unmanned aerial vehicles (UAVs) in precision agriculture [9,10,11]. A few studies have used them for potato growth monitoring and trait estimation. For example, UAV-based RGB imagery was used for potato identification [12] and emergence estimation [13]. The leaf chlorophyll content of potato was estimated using multispectral data [14].

Compared with other imaging modalities, such as natural color and multispectral imagey, hyperspectral imagery consists of hundreds of spectral bands, arranged in a narrower bandwidth, and is thus capable of providing more detailed spectral information which is of great importance for estimating complex plant traits, such as yield [15]. For example, the grain yield of paddy fields was predicted in the northern part of Vientiane in Laos, and the booting stage was found to be the best prediction time using hyperspectral data [16]. Also, wheat yield was successfully estimated using 190 hyperspectral narrow bands acquired from a UAV platform before harvest in Minnesota [17]. Through comparative experiments, hyperspectral imagery was validated to be more effective than RGB imagery in modeling the variability in winter barley yield [18]. Though successful, most studies have concentrated on cereal crops, and it is essential to exploit the potential of applying hyperspectral imaging to model tuber production in potatoes.

In general, biophysical crop simulation models and statistical machine learning models are the two main types of approaches for crop yield prediction. The simulation models forecast crop productivity by combining crop growth information including the physiological characteristics of plants, nutrient cycling, and environmental factors [19]. Over the past few decades, several simulation models have been developed for potato, such as POMOD [20], AQUACROP [21], APSIM-Potato [22], PotatoSoilWat [23], and LINTUL-Potato [24]. Although powerful, they typically require extensive crop-specific data as inputs, such as crop variety, management, and soil conditions, which are often difficult to obtain. Moreover, it can also be challenging to calibrate these mechanistic models due to the uncertainty of input data and the complexity of the physiological processes [25]. On the other hand, data-driven machine learning methods aim to model the empirical relationship between the input variables and the crop productivity. Unlike the crop simulation models, these approaches are not directly based on the physiological mechanisms of plant growth, and thus can forecast the crop production without depending on the specific factors for individual crops. For example, Gomez et al. developed machine learning models for in-season potato yield prediction in Spain, using multispectral satellite images [26].

This study was designed to perform in-season prediction of end-of-season tuber production under four different irrigation treatments, using UAV-based hyperspectral images and machine learning. Specifically, six mainstream machine learning models, i.e., ordinary least squares (OLS), ridge regression, partial least square regression (PLSR), SVR, RF, and adaptive boosting (AdaBoost), were built and compared for modeling two important potato traits, tuber yield, and tuber set (the total number of tubers produced per unit). The specific objectives included (1) investigating the potential of using hyperspectral images for potato yield and tuber set prediction, (2) comparing different model performances for each trait prediction, and (3) evaluating the model adaptability under different irrigation treatments.

## 2. Materials and Methods

### 2.1. Experimental Design and Field Data Collection

**Figure 1:**
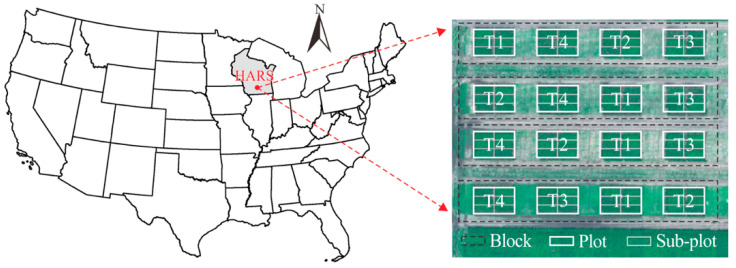
Experimental site location. T1, T2, T3 and T4 represent four irrigation rates: 50%, 75%, 100% and 125% of actual crop evapotranspiration (ET_c_).

The field experiments were conducted at the University of Wisconsin Hancock Agricultural Research Station (HARS) under a Variable Rate Irrigation (VRI) system in Hancock, WI, USA (Figure 1) in the 2019 growing season. Wisconsin is located in the Great Lakes region which is in the north-central part of the United States, and Hancock is located in central Wisconsin. In 2019, Hancock experienced a typical humid continental climate with an average 23 °C daytime temperature and 12 °C nighttime temperature during the growing season from late April to early September [27]. The monthly total precipitation was the highest at 18 cm in July and the lowest at 7 cm in August.

For the field trials, six potato varieties were evaluated under four irrigation treatments. Planting occurred on May 1st, and harvesting took place on September 24th. Both planting and harvesting were conducted using customized machines. The experimental design was a split plot design with four replications, with the whole plot as the irrigation treatment and the sub-plot as six different potato varieties. The size of each sub-plot was 7.6 m long and 1.8 m wide, and there were 50 plants in each sub-plot. The four irrigation rates included 50% of ET_c_ (under-irrigation), 75% of ET_c_ (under-irrigation), 100% of ET_c_ (standard irrigation), and 125% of ET_c_ (over-irrigation). ET_c_ refers to the actual crop evapotranspiration (ET), which is calculated by potential ET × ground cover %. Potential ET was obtained daily from the UW Extension Ag Weather Mailer, and ground cover was determined by the CANOPEO app published by the Oklahoma State University [28]. ET_c_ was adjusted before ground cover reached 80%. Once 80% cover was reached, ET_c_ = potential ET. At the final harvest, tuber yield was measured by weighing each individual tuber from every sub-plot, and tuber set was counted as the total number of tubers in each sub-plot. The detailed tuber yield and tuber set statistics (minium value (Min.), mean value (Mean), maximum value (Max.) and standard diviation (STD)) for each irrigation treatment are shown in Table 1 and Table 2, respectively, and lower production was observed when the field was under-irrigated.

### 2.2. UAV-Based Hyperspectral Image Acquisition and Preprocessing

The hyperspectral data were acquired by a Headwall nano-hyperspec imager that offers 273 spectral bands ranging from 400 nm to 1000 nm with a bandwidth at 2.2 nm. A lightweight navigation system was deployed on a hyperspectral camera to directly provide the absolute location of the camera for data geo-referencing. The camera was mounted on a DJI Matrice 600 Pro (DJI Technology Co., Shenzhen, China) UAV platform with a three-axis gimbal stabilizer. The UAV flights were conducted on August 6th and 15th in 2019 under low wind speed and clear sky conditions. Those dates were picked because they were during the tuber maturation stage, suggesting more accurate prediction as they were close to the final harvest date compared to timings early in the growing season. The flight mission was conducted at a height of 40 m with a speed of 5 m/s, resulting in a 2.5 cm ground sampling distance (GSD).

After the data acquisition, two main preprocessing operations were needed. For the geometric correction, SpectralView software (Headwall photonics, Inc., Boston, MA, USA) was employed to orthorectify the raw hyperspectral data based on the GNSS/INS data. For the radiometric correction, the raw digital numbers (DN) in the hyperspectral images were first converted to radiance and then radiometrically calibrated to reflectance using the reference panels with 56%, 32%, and 11% reflectivity. After geometric and radiometric correction, the background (e.g., shadow and soil) in each sub-plot needed to be removed. In this study, an NIR band at 800 nm wavelength was selected as a threshold, and the reflectance value of the pixels below 45% was regarded as the background. The preprocessed data were extracted from each split-plot, and the mean spectrum of each split-plot was used to represent the plot spectral information.

### 2.3. Model Development and Performance Evaluation

In this study, we developed models to predict tuber yield and tuber set using the hyperspectral data acquired at different times. Each sub-plot was considered a data sample. The dependent variable was the yield (or tuber set) acquired from the end-of-season harvest. The spectral data acquired from the two flights were used both independently and then combined as the independent variables. Six mainstream machine learning methods were explored in this study, and a brief introduction of these algorithms is presented below.

OLS is a commonly used linear regression algorithm that aims to minimize the sum of squared errors between the observed and predicted values. However, it is subjected to overfitting with a large number of input features, since penalties on the coefficients are lacking. To overcome this issue, Ridge was proposed by adding an L2 norm regularization based on the original least square loss function in OLS to avoid large coefficients and thus help reduce overfitting [29]. The PLSR is a statistical method that projects both the predicted and the observed variables to a new space and finds a linear regression model in the new space. The projection helps reduce the dimensionality of the input data, and it is achieved by using a technique similar to principal components analysis [30]. SVR is an effective supervised learning approach with good generalization capability. In SVR, the first step is to map the input variables from the original dimensional space to a high-dimensional feature space through a kernel function [31]. Second, a linear model is constructed in the new feature space to fit the data. The kernel function can be either linear or non-linear depending on the input variables, and a radial basis function (RBF) kernel was adopted in this study. RF is one of the ensemble learning algorithms which consists of a large number of individual decision trees [32,33]. The final result is obtained by averaging the predictions from all individual trees. AdaBoost is another ensemble learning method that uses the boosting strategy to generate each individual tree in a sequential way, and the weighted average method is used to obtain the final prediction [34].

We implemented all the machine learning algorithms in Python. To validate the robustness of the developed models, we adopted a four-fold cross validation strategy for both tuber yield and tuber set predictions. Specifically, all the input variables were randomly and evenly divided into four subsets. In each round, one of the four subsets was used as the test set while the remaining three subsets were treated as the training set. The coefficient of determination (R^2^) and root mean square error (RMSE) metrics were used for evaluating the model performance in each round. The final prediction accuracies were obtained by averaging the results from the four rounds.

## 3. Results and Discussion

### 3.1. Model Comparison and Performance

The six machine learning models described in Section 2 were trained independently for modeling the tuber yield and tuber set using the single-day data acquired on August 6th and August 15th and also the combined data of the two days, and their accuracies are reported in Table 3 and Table 4, respectively. In general, the combined data improved the model performance compared with single-day data, which was likely because the combined data included more growth information of potato during the tuber-bulking period. Therefore, the following analysis was based on the results from using the two-day combined data.

**Figure 2:**
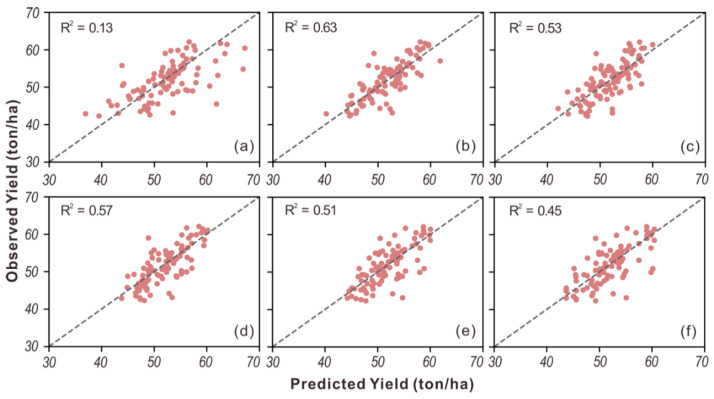
The scatter plots of observed vs. predicted tuber yield from (**a**) OLS, (**b**) Ridge, (**c**) PLSR, (**d**) SVR, (**e**) RF, and (**f**) AdaBoost.

**Figure 3:**
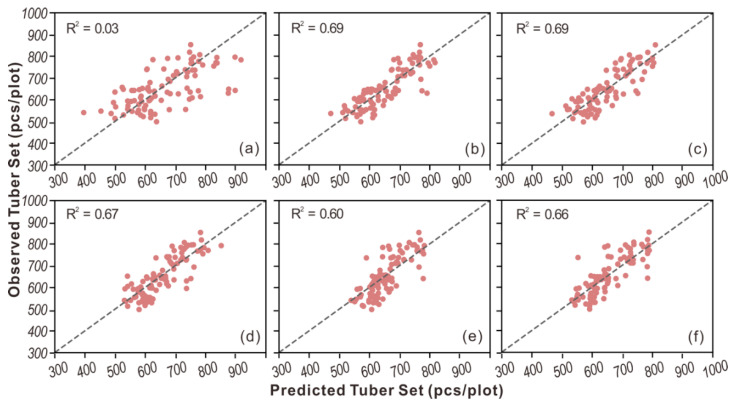
The scatter plots of observed vs. predicted tuber set from (**a**) OLS, (**b**) Ridge, (**c**) PLSR, (**d**) SVR, (**e**) RF, and (**f**) AdaBoost.

For the tuber yield, the Ridge approach achieved the best performance with an R^2^ of 0.63, while the other five models had R^2^ less than 0.60. For the tuber set, similar prediction accuracies were obtained by Ridge and PLSR, achieving an R^2^ of 0.69, followed by SVR and AdaBoost. Also, we found that OLS performed the worst and obtained much lower accuracies than others. This is mainly because OLS is a simple linear model and lacks penalties for the coefficients, thus causing overfitting when there is a large number of input variables. Besides, we also noticed that the tuber set could be better predicted than the tuber yield. This is primarily because the tuber set is closely related to the aboveground stems which can be directly measured by the spectral data [35], whereas there is no clear relationship between aboveground biomass and tuber growth underground. In addition, the agreement between the observed and the predicted tuber yield for each model is shown in Figure 2, and similar scatter plots for tuber set are shown in Figure 3. Among the six models, the best agreement was found for the Ridge model for tuber yield (Figure 2b). Ridge and PLSR showed better agreement than other models for the tuber set, (Figure 3b,c). In contrast, the worst agreement was observed for OLS for both tuber yield (Figure 2a) and tuber set (Figure 3a).

We further analyzed the model performance based on the characteristics of the six models. Ridge regression shrinks the coefficients by introducing squared regularization term [36] and hence effectively reduces the sensitivity to outliers and negative effects of multi-collinearity [37]. The superiority of ridge regression in using high dimensional data was observed in this study and also in several other studies on hyperspectral-based crop yield prediction [38,39]. PLSR and SVR are commonly used algorithms for processing high dimensional inputs, and they have distinct principles. Specifically, PLSR searches for a group of low-dimensional latent vectors and performs regression based on these components [40], while SVR projects the inputs into a higher dimensional space and constructs a linear model for regression [41]. They were second only to Ridge in tuber yield and tuber set prediction. Both RF and Adaboost are tree-based ensemble learning techniques, and they are widely used in the field of crop yield prediction [5,42]. However, in this study, they were found to be inferior to Ridge, PLSR, and SVR in dealing with a small sample size with high dimensional features. Although OLS is intuitively understandable and computationally efficient, it failed to process complex high dimensional data. As OLS performed the worst among all the approaches for both yield and tuber set prediction, we excluded it from the following analysis.

### 3.2. Model Adaptability Under Different Irrigation Treatments

**Figure 4:**
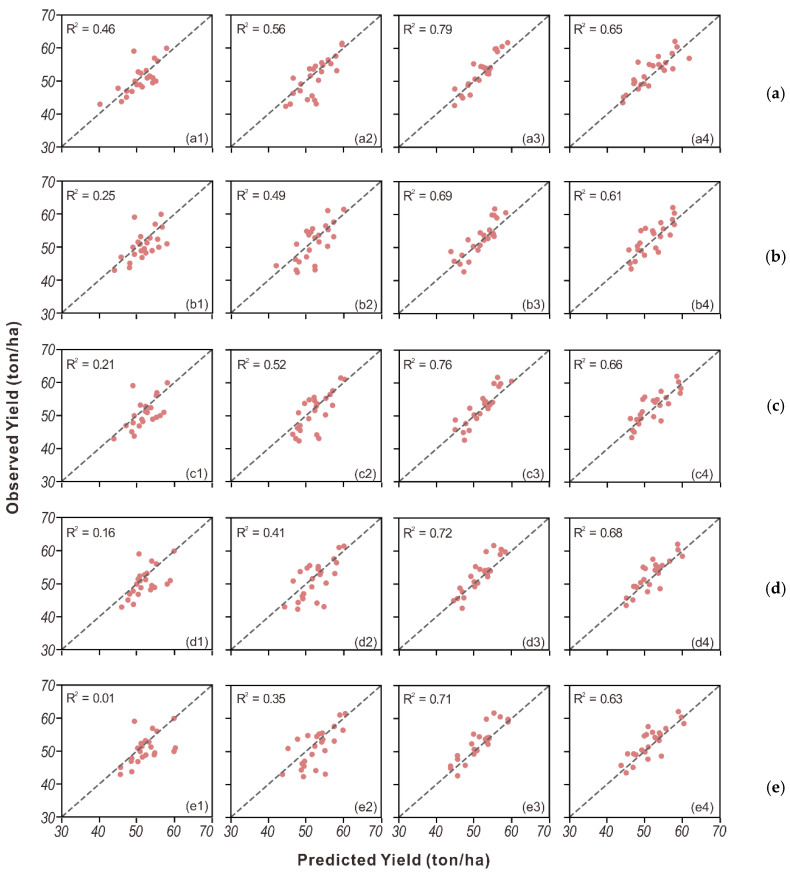
Scatter plots of observed vs. predicted yield from (**a**) Ridge, (**b**) PLSR, (**c**) SVR, (**d**) RF and (**e**) AdaBoost for groups with four different irrigation treatments: (1–4) 50% of ET_c_, 75% of ET_c_, 100% of ET_c_, and 125% of ET_c_.

**Figure 5:**
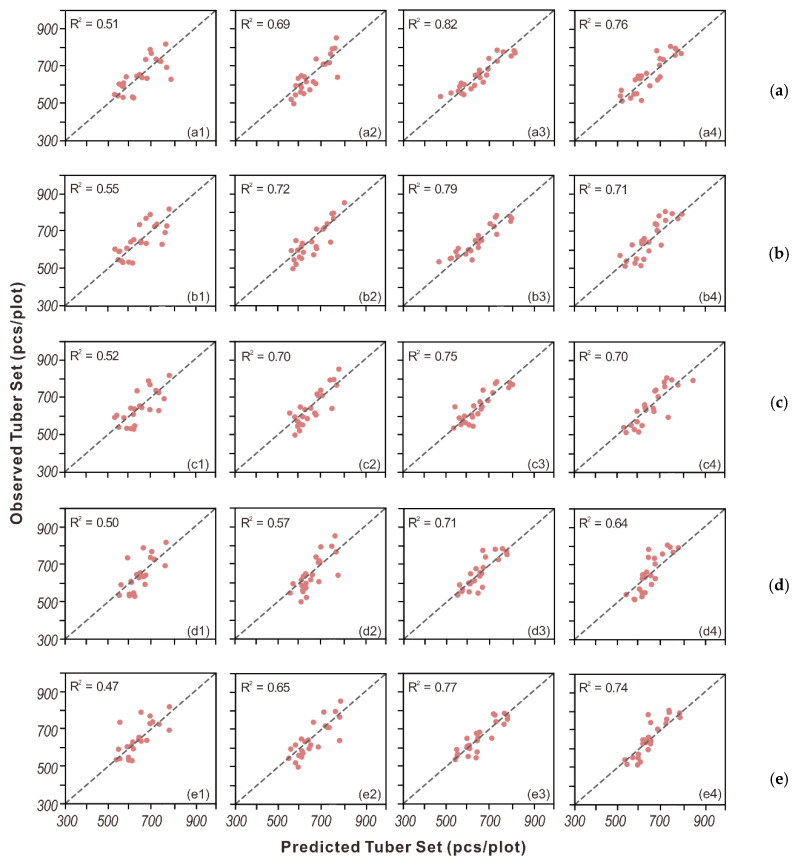
Scatter plots of observed vs. predicted tuber set from (**a**) Ridge, (**b**) PLSR, (**c**) SVR, (**d**) RF, and (**e**) AdaBoost for groups with four different irrigation treatments: (1–4) 50% of ET_c_, 75% of ET_c_, 100% of ET_c_, and 125% of ET_c_.

Besides the overall performance, the model adaptability under the four irrigation treatments was also evaluated. For this purpose, the prediction accuracy for each treatment was reported for tuber yield in Table 5 and tuber set in Table 6. Generally, the best prediction accuracy was obtained in the 100% ET_c_ treatment, followed by 125% ET_c_, and the lowest accuracy was observed in the 50% ET_c_ treatment. For the tuber yield, the Ridge model performed the best for most treatments except for 125% ET_c_. For the tuber set, PLSR had a better performance for under-irrigation rates, with an R^2^ of 0.55 and 0.72 for 50% ET_c_ and 75% ET_c,_ respectively, and the Ridge model achieved better performance for the standard and over-irrigation rates, achieving an R^2^ of 0.82 and 0.76, respectively. Finally, the detailed tuber yield and tuber set modeling performance for each treatment are shown in scatter plots in Figure 4 and Figure 5. It is clear to see the best agreement was achieved in the 100% ET_c_ treatment in both tuber yield and tuber set prediction across all approaches. Additionally, strong and stable agreement was demonstrated in Ridge for tuber yield and tuber set modeling.

### 3.3. Analysis of Feature Importance

To explore which hyperspectral band is important for predicting the tuber yield and tuber set, we computed the feature importance using the Gini index which has been widely adopted in tree-based models (Random Forest and AdaBoost) [43,44,45].

**Figure 6:**
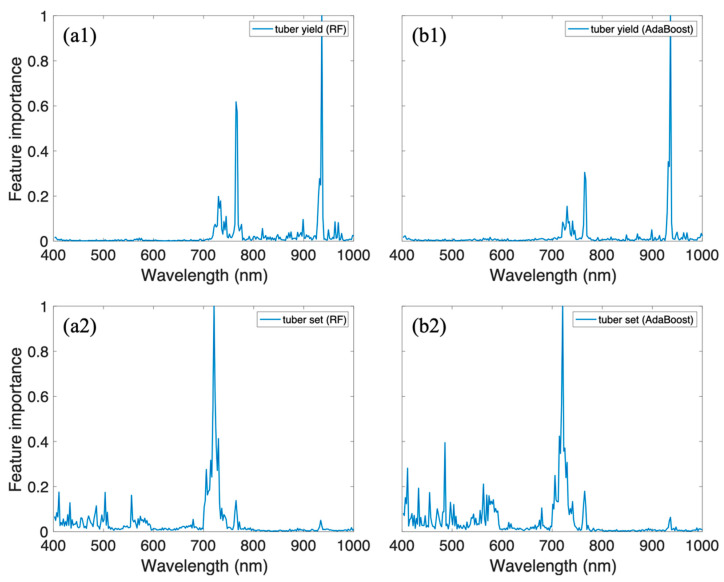
Feature importance of RF for (**a1**) tuber yield; (**a2**) tuber set; Feature importance of AdaBoost for (**b1**) tuber yield and (**b2**) tuber set.

From Figure 6a1,b1, we can find that the values of feature importance around 780 nm and 950 nm were extremely higher than others. For tuber set, the most important wavelength was different from that predicting tuber yield (Figure 6a2,b2). Specifically, the bands around 720 nm, 500 nm, and 550 nm were more significant than other spectral bands. A similar pattern was observed that the red edge is important for both tuber yield and tuber set prediction, which is consistent with previous research where the red edge was included in the vegetation indices and successfully predicted crop yield [42]. In this study, all the spectral bands were used for model development. In future work, we will investigate the band selection method to further enhance the prediction performance.

## 4. Conclusions

In this study, we explored six mainstream machine learning models, OLS, Ridge, PLSR, SVR, RF, and AdaBoost, for potato tuber yield and tuber set predictions using UAV-based hyperspectral imagery. The spectral data acquired on August 6th and 15th were combined for the model development, and the prediction accuracy was much better than using the single-day data. Using the combined data, the Ridge model performed best among all six models in tuber yield modeling, and Ridge and PLSR outperformed other models in tuber set modeling. Additionally, we evaluated the model performance under four irrigation treatments including two under-irrigation rates (50% of ET_c_ and 75% of ET_c_), a standard irrigation rate (100% of ET_c_), and an over-irrigation rate (125% of ET_c_), and again, Ridge achieved the best performance in tuber yield prediction for most treatments, and Ridge and PLSR performed better in tuber set prediction than other models across treatments.

Unlike other crops, potatoes are stem tubers which are situated beneath the surface of the ground throughout the growing season. Using the UAV remote sensing, we acquired potato hyperspectral data by imaging the surface of leaves which are directly connected to the belowground potato tubers through nutrient transportation within the stems [46]. The results of this study demonstrated the feasibility and potential of using in-season hyperspectral imagery and the machine learning technique for predicting end-of-season tuber yield and tuber set. The advanced sensing and prediction methods can also potentially be applied to other tuber crops, such as sweet potatoes, cassavas, and yams, which grow underground like potatoes. To further improve the prediction performance, new research is underway to acquire time series hyperspectral images at each growth stage of the potato crop, consecutively measured soil moisture content at different soil depths throughout the growing season, and weather factors such as daily average temperature, precipitation, solar radiation, and wind speed to be incorporated into the modeling process. Precisely and timely estimation of potato production using in-season information can help potato growers optimize their irrigation management to achieve long-term water use sustainability while reducing the risk of yield and quality loss caused by water stress.

## Figures and Tables

**Table 1:** Tuber yield statistics under four irrigation treatments.

Irrigation Rate	Min. (ton/ha)	Mean (ton/ha)	Max. (ton/ha)	STD (ton/ha)
50% of ET_c_	42.92	50.57	59.89	4.32
75% of ET_c_	42.33	51.15	61.43	5.53
100% of ET_c_	42.62	52.62	61.70	5.09
125% of ET_c_	43.51	52.63	62.09	4.76

**Table 2:** Tuber set statistics under four irrigation treatments.

Irrigation Rate	Min. (pcs/plot)	Mean (pcs/plot)	Max. (pcs/plot)	STD (pcs/plot)
50% of ET_c_	530	650	819	85
75% of ET_c_	499	647	853	92
100% of ET_c_	537	653	786	81
125% of ET_c_	514	657	807	96

**Table 3:** The performance of the six machine learning models for tuber yield prediction.

Model	Metrics	Aug. 6 + Aug. 15	Aug. 6	Aug. 15
OLS	R^2^	0.13 (0.26)	−0.51 (0.79)	−0.45 (0.67)
	RMSE	4.53 (0.33)	5.85 (1.22)	5.81 (1.42)
Ridge	R^2^	0.63 (0.03)	0.58 (0.12)	0.55 (0.07)
	RMSE	3.03 (0.29)	3.21 (0.57)	3.33 (0.31)
PLSR	R^2^	0.53 (0.04)	0.37 (0.09)	0.53 (0.06)
	RMSE	3.40 (0.24)	3.92 (0.25)	3.40 (0.29)
SVR	R^2^	0.57 (0.03)	0.39 (0.11)	0.52 (0.03)
	RMSE	3.28 (0.28)	3.90 (0.57)	3.47 (0.32)
RF	R^2^	0.51 (0.10)	0.45 (0.03)	0.43 (0.13)
	RMSE	3.46 (0.28)	3.70 (0.41)	3.73 (0.30)
AdaBoost	R^2^	0.45 (0.08)	0.46 (0.06)	0.38 (0.14)
	RMSE	3.66 (0.29)	3.66 (0.49)	3.87 (0.42)

**Table 4:** The performance of the six machine learning models for tuber set prediction.

Model	Metrics	Aug. 6 + Aug. 15	Aug. 6	Aug. 15
OLS	R^2^	0.03 (0.30)	−2.99 (0.86)	−0.49 (1.39)
	RMSE	85.27 (14.09)	175.01 (24.09)	96.71 (40.59)
Ridge	R^2^	0.69 (0.10)	0.61 (0.09)	0.61 (0.12)
	RMSE	48.15 (6.72)	54.77 (7.36)	54.50 (6.69)
PLSR	R^2^	0.69 (0.07)	0.61 (0.11)	0.57 (0.15)
	RMSE	48.81 (4.40)	54.96 (8.70)	56.44 (8.34)
SVR	R^2^	0.67 (0.05)	0.55 (0.06)	0.60 (0.06)
	RMSE	50.65 (5.04)	58.66 (4.87)	55.80 (4.29)
RF	R^2^	0.60 (0.08)	0.45 (0.13)	0.59 (0.06)
	RMSE	55.62 (6.39)	64.71 (8.09)	56.34 (5.20)
AdaBoost	R^2^	0.66 (0.08)	0.45 (0.27)	0.56 (0.02)
	RMSE	50.97 (7.93)	63.31 (15.37)	58.28 (3.04)

**Table 5:** Performance of five machine learning models in tuber yield prediction under four irrigation treatments.

Model	Metrics	50% of ET_c_	75% of ET_c_	100% of ET_c_	125% of ET_c_
Ridge	R^2^	0.46	0.56	0.79	0.65
	RMSE	3.16	3.68	2.36	2.82
PLSR	R^2^	0.25	0.49	0.69	0.61
	RMSE	3.73	3.95	2.84	2.98
SVR	R^2^	0.21	0.52	0.76	0.66
	RMSE	3.83	3.85	2.51	2.79
RF	R^2^	0.16	0.41	0.72	0.68
	RMSE	3.96	4.26	2.71	2.68
AdaBoost	R^2^	0.01	0.35	0.71	0.63
	RMSE	4.30	4.45	2.75	2.89

**Table 6:** Performance of five machine learning models in tuber set prediction under four irrigation treatments.

Model	Metrics	50% of ET_c_	75% of ET_c_	100% of ET_c_	125% of ET_c_
Ridge	R^2^	0.51	0.69	0.82	0.76
	RMSE	59.21	51.17	34.28	46.48
PLSR	R^2^	0.55	0.72	0.79	0.71
	RMSE	57.09	48.39	37.34	51.21
SVR	R^2^	0.52	0.70	0.75	0.70
	RMSE	58.68	50.79	40.63	52.17
RF	R^2^	0.50	0.57	0.71	0.64
	RMSE	60.23	60.69	43.89	57.55
AdaBoost	R^2^	0.47	0.65	0.77	0.74
	RMSE	61.65	54.59	38.78	49.15
